# Seed-coating of rapeseed (*Brassica napus*) with the neonicotinoid clothianidin affects behaviour of red mason bees (*Osmia bicornis*) and pollination of strawberry flowers (*Fragaria × ananassa*)

**DOI:** 10.1371/journal.pone.0273851

**Published:** 2022-09-08

**Authors:** Lina Herbertsson, Björn K. Klatt, Maria Blasi, Maj Rundlöf, Henrik G. Smith

**Affiliations:** 1 Centre for Environmental and Climate Research, Lund University, Lund, Sweden; 2 Department of Biology, Lund University, Lund, Sweden; University of Leipzig Faculty of Life Sciences: Universitat Leipzig Fakultat fur Lebenswissenschaften, GERMANY

## Abstract

Neonicotinoid insecticides applied to flowering crops can have negative impacts on bees, with implications for crop pollination. To assess if exposure to the neonicotinoid clothianidin via a treated crop (rapeseed) affected bee behaviour, pollination performance (to strawberry), and bee reproduction, we provided each of 12 outdoor cages with rapeseed (autumn-sown plants complemented with a few spring-sown plants to extend the flowering period) grown from either clothianidin-treated or untreated (control) seeds, together with strawberry plants and a small population of red mason bees (*Osmia bicornis*). We expected clothianidin to reduce bee foraging activity, resulting in impaired strawberry pollination and bee reproduction. During the early stage of the experiment, we observed no difference between treatments in the length of entire foraging trips, or the combined number of rapeseed and strawberry flowers that the bees visited during these trips. During the later stage of the experiment, we instead determined the time a female took to visit 10 rapeseed flowers, as a proxy for foraging performance. We found that they were 10% slower in clothianidin cages. Strawberries weighed less in clothianidin cages, suggesting reduced pollination performance, but we were unable to relate this to reduced foraging activity, because the strawberry flowers received equally many visits in the two treatments. Clothianidin-exposed females sealed their nests less often, but offspring number, sex ratio and weight were similar between treatments. Observed effects on bee behaviour appeared by the end of the experiment, possibly because of accumulated effects of exposure, reduced bee longevity, or higher sensitivity of the protocols we used during the later phase of the experiment. Although the lack of a mechanistic explanation calls for interpreting the results with cautiousness, the lower strawberry weight in clothianidin cages highlights the importance of understanding complex effects of plant protection products, which could have wider consequences than those on directly exposed organisms.

## Introduction

Wild and managed bees are important providers of pollination services and benefit the production of various insect-pollinated crops [1, 2]. Recent bee declines are thought to be driven by several interacting factors, many related to agricultural intensification [3]. The use of insecticides, which is an integral part of contemporary agriculture, is expected to be one of them [4]. Neonicotinoids are a group of insecticides, which is associated with reduced bee reproduction [5, 6] and declining wild bee populations [7], potentially by distorting the bees’ foraging behaviour [8–10] and navigation [11, 12]. Because of concerns about bees and crop pollination, the outdoor use of three neonicotinoids for plant protection is now restricted in the European Union, but in many other countries these compounds are still being used [13].

Most of the studies on how neonicotinoids affect bees have focused on either bumblebees or honeybees [14, 15], and despite concerns that neonicotinoids can threaten pollination services [16], there are few empirical studies investigating their impact on crop pollination [but see 17, 18].

We tested if exposure via a treated crop (*Brassica napus*) alters the foraging behaviour of a solitary bee, *Osmia bicornis*, and if this translates into secondary impacts on pollination services in an adjacent crop (*Fragaria × ananassa*). To study this, we performed a replicated experiment in 12 outdoor cages, each containing a small population of *O*. *bicornis*. In each cage, there were 10 untreated strawberry plants and 11 rapeseed plants, grown from either uncoated seeds (control cages) or seeds coated with the neonicotinoid clothianidin (Elado®, Bayer Crop Science) (clothianidin cages). We expected that exposure to treated rapeseed would reduce bee foraging activity, resulting in fewer visits to strawberry flowers and thereby reduced strawberry pollination, revealed by lower strawberry weight. In addition, we expected that anticipated behavioural aberrations would negatively affect reproduction, with possible impact on population persistence and thus future pollination services.

## Materials and methods

### Study organisms

*Osmia bicornis* is a generalist forager bee [19]. It forages on rapeseed [20] as well as strawberry flowers [21] and is considered a suitable pollinator of strawberry flowers [22]. The species nests in cavities above ground, where females construct series of brood cells (from the inner part of the cavity to the entrance) which they provide with pollen before they lay an egg and seal the cell with mud. After finishing and sealing the last cell of a cavity, the female commonly seals the outer part of the cavity with an end mud plug [19].

Strawberries are aggregated fruits, with the true fruits being the nuts (so-called achenes) on the surface of the strawberry [23]. Fertilized achenes produce auxin which induces strawberry growth [24] and an increasing number of fertilized achenes therefore results in higher strawberry weight [25]. Most strawberry varieties are self-fertile, but stigmas and anthers are spatially separated, and in addition, stigmas are receptive before pollen is released [23]. Insects therefore contribute to pollination, seemingly by dispersing pollen among as well as within flowers [26]. Consequently, strawberry weight benefits from an increasing number of bee visits, up to around 60 visits [23]. Flowers grow in clusters and the primary flower of each branch, which is the first one to open, contains more ovules than later ones, resulting in the largest and commercially most important strawberry [23].

### Experimental setup

**Fig 1:**
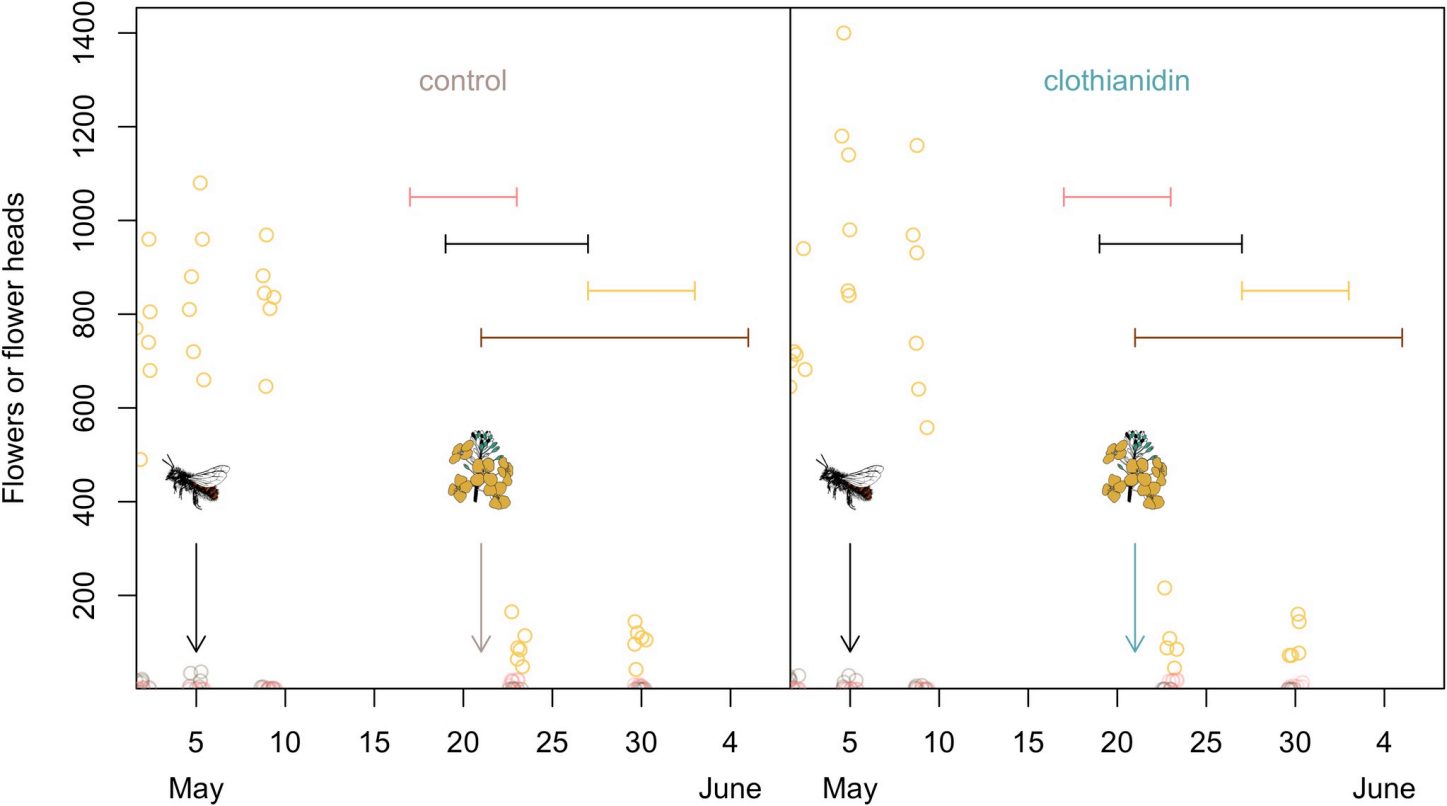
The circles indicate the number of open rapeseed (yellow) and strawberry (red) flowers, or flower heads of dandelion (brown), which we measured in all the cages on the 2^nd^, 5^th^, 9^th^, 23^rd^ and 30^th^ May. The vertical arrows indicate the day when we manually released the females that had not emerged from their cocoons (black) and the day when two untreated (beige) or treated (turquoise) spring-sown rapeseed plants were added to cages. The horizontal solid lines indicate the periods during which data were collected for flower-visitation of strawberry plants (red), entire foraging trips (black) and ten visits to rapeseed flowers (yellow). The brown horizontal line indicates the period, during which we observed new nest plugs appearing.

In each of 12 outdoor cages, we placed 10 strawberry plants (variety ‘Honeoye’, Kraege, Germany) and 11 rapeseed plants (9 plants of the autumn-sown variety ‘Visby’ and 2 plants of the spring-sown variety ‘Majong’). The plants were potted individually in 10 L pots. We irrigated the plants when needed with similar amount of water for all plants of the same crop species, which we ensured by watering them with a hose for the same number of seconds. We also provided each cage with 9 cocoons (4 females and 5 males) of *O*. *bicornis*, a wooden trap nest with 80 holes (8 mm Ø), and a plastic box (25 ×18 × 7 cm) with around 2 L of mud. Cages were cubic, (1.80 × 1.80 × 1.80 m), and consisted of a wooden frame, where the sides and the roof were covered with mesh (gap size: 0.25 mm). The ground was covered with grass and a few dandelions. Before releasing the bees into the cages, we removed all flower heads and buds of dandelion (*Taraxacum officinale*). To minimize disturbance, we did not enter the cages to remove dandelions after releasing the bees, but dandelions were scarce during data collection (Fig 1).

Autumn-sown rapeseed plants originated from an experimental crop field (experiment conducted by the industry company Svensk Raps AB (Swedish Rapeseed Inc.)), which had been sown with 16 intermixed plots (1.62 × 8.0 m) of seeds that were either uncoated or coated with Elado® (Bayer Crop Science, 10 gram clothianidin + 2 gram ß-cyfluthrin per kg seeds). We dug up seven plants per plot in the middle of March. The chosen plants were all from the edges of the plots and never adjacent to each other. We transferred them with the closest soil around the roots and planted them in potting soil. The plants were kept in the cages until we set up the experiment and we then divided them equally among the cages, aiming to keep the standard of the plants as similar as possible among cages.

For the spring-sown variety, seed treatment with Elado® (Bayer Crop Science, 10 gram clothianidin + 2 gram ß-cyfluthrin per kg seeds) was conducted by the Rural Economic and Agricultural Society according to instructions from the manufacturer (Bayer Crop Science). We grew spring-sown rapeseed plants in a greenhouse, simulating Swedish spring (20–25°C, 14h/10h artificial light/darkness), and added them to the cages the 21^st^ of May. We did this to enhance the availability of food and ensure continued exposure to clothianidin in the clothianidin cages after the autumn-sown rapeseed had peaked flowering (Fig 1).

In six (clothianidin) cages, we used rapeseed grown from the coated seeds, and in six (control) cages, we used rapeseed grown from uncoated seeds. Since clothianidin [27], but not cyfluthrin [28], is systemic, we expected flower-visiting bees to only be exposed to clothianidin (not cyflythrin). In another study, we verified that bees were not exposed to cyfluthrin when foraging on rapeseed with the same seed treatment [6]. We placed the rapeseed plants in the cages by mid-March. A month later, we potted the strawberry plants and added them to the cages. To guarantee co-flowering of the two crops, we advanced the flowering time for half of the strawberry plants, which we moved from the cages to a greenhouse for two weeks. These plants were sprayed once with pyrethrin (NatriaPyrsol®, Bayer, 0.045 g A.I. per L), following the instructions for application on the container, to treat against *Thrips sp*. in the greenhouse. Because pyrethrin has a short half-life when exposed to sunlight [29], we kept the plants outdoors for a week before reintroducing them to the cages to avoid that the bees became exposed to high levels of pyrethrin. We cannot guarantee that the bees were unexposed to pyrethrin, but the treated plants were evenly distributed among the cages such that any exposure would be equal between treatments.

We purchased cocoons from a commercial supplier (Dr. Schubert Pflanzenzucht, Germany) and distinguished between male (bright face) and female (dark face) bees by opening the front plug of the cocoon. The cocoons of each sex were randomly sorted into 12 equally large groups, one for each cage and we placed them in the cages the 25^th^ of April. All males emerged the same day and the females emerged sporadically during the following 10 days. In eight of the cages (four per treatment), a total of 13 females across cages had not yet emerged the 5^th^ of May and were then manually released from the cocoons (Fig 1, S1 Table). A few small male non-*Osmia* bees that emerged from the ground in six cages—later confirmed as being three within each treatment—were left in the cages, because we avoided opening the cages when the bees were active. These bees visited both rapeseed and strawberry flowers, but since male bees do not collect pollen [30] they were expected to have little impact compared to the females of *O*. *bicornis* (which was later confirmed, see Results). During strawberry harvest, the 10^th^ of June, we observed a pollen-collecting non-*Osmia* female, which we immediately caught and released outside the cage. We expected a pollen-collecting individual in only one of the cages to be a possible source of bias and catching it was an easy way of avoiding that, as we were already in the cage to collect strawberries. However, we harvested the last strawberries 5^th^ July and because it takes around a month for strawberries to ripen [31], this decision, likely had no impact on the results. In most cages (four clothianidin and five control), we observed a few ants on the strawberry flowers and on rare occasions, we observed small wasps (one clothianidin and one control), a spider (control) and a fly (clothianidin) on open flowers. We decided to leave them in the cages. All the above decisions were taken without knowledge of the treatment of individual cages.

### Data collection

All assessments of bee behaviour were performed from outside of the cage. Usually between two and six people collected data simultaneously, which allowed us to collect data from the two treatments at the same time. When a certain measurement had been taken, the data collector continued to another cage until she or he had collected data from all the cages. Most data collectors (five out of six) were blinded to the treatment of each cage during the whole experiment. One data collector was, for practical reasons, aware of cage treatment from 21^st^ May when the spring-sown rapeseed plants were added to the cages.

**Fig 2:**
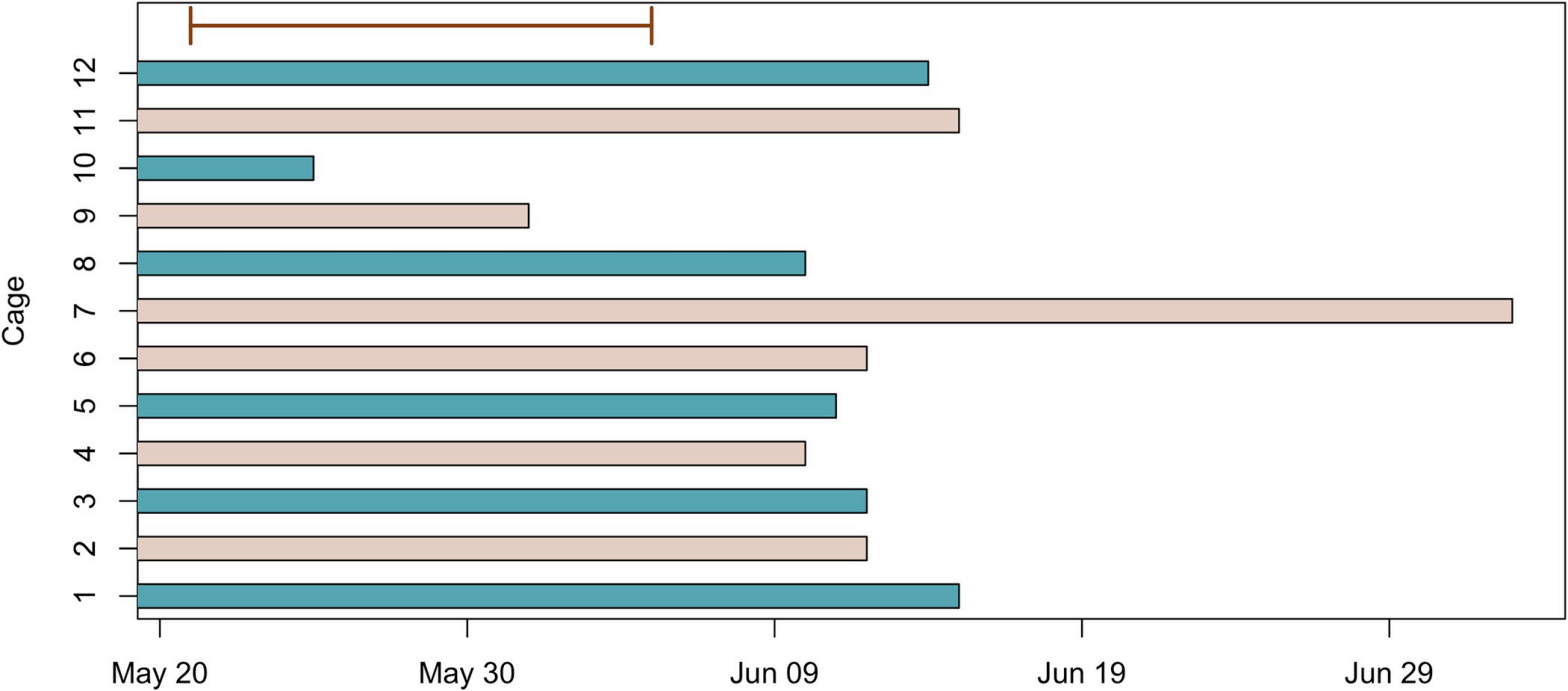
The bars indicate for how long bees were observed alive in each of the control (beige) and clothianidin (turquoise) cages. The brown line indicates the period, during which we observed new nest plugs appearing.

Observations of entire foraging trips were conducted between 19^th^ and 27^th^ May (Fig 1). For each cage, we followed the first *O*. *bicornis* female leaving the trap nest until it returned to the nest. We measured the time the bee used to accomplish an entire foraging trip and counted the number of flower visits per trip. Since this method turned out to be very time-consuming, we switched to another observation method. Between 27^th^ May and 2^nd^ June, we instead picked the first observed foraging *O*. *bicornis* female and measured the time it used to accomplish 10 flower visits (Fig 1). We did not observe any other activities, such as mud collection or resting, during these observations. To reduce unexplained variation, we later excluded sequences where the bee visited any other flowers than rapeseed because 98.1 ± 0.8% (raw mean ± sd) of the flower visits were to this crop. All males had died at this time and female bees had started to build nests. We conducted observations between 9:00 am and 4:30 pm. Weather conditions varied between observation days, but no observations were conducted in rainy or stormy weather. We took 20 to 27 measurements per cage (except for one clothianidin cage where all bees were dead at that time (Fig 2)) on the time it took for a female to visit 10 rapeseed flowers.

Between 17^th^ and 23^rd^ May, we performed focal observations of strawberry plants, counting all bee visits to any of the open strawberry flowers for 2 minutes (Fig 1). Occasionally, data collectors missed that the 2 minutes had passed and then concluded the observation for that cage after the next half-minute had passed (i.e., after 2:30 or 3:00 minutes). To equalize the observation times among cages, the observer then prolonged the time for all next-coming cages in that observation round and if needed (i.e., if this was not the first cage of the round) for the previously observed cages in the next round, so that all cages had been observed equally long every day. The total observation time was 74 min per cage, divided into 34 separate sessions, during 5 different days. Data were collected between 1:00 and 4:30 pm under sunny and warm weather conditions. At each but one day of observations, we also counted the number of open strawberry flowers per cage; for the day with missing data (22^nd^ May) we instead calculated the mean number of open flowers during the preceding and following days. In addition, at five occasions (2^nd^, 5^th^, 9^th^, 23^rd^ and 30^th^ May), we counted all open flowers of rapeseed, strawberry, and dandelion in the cages (Fig 1). These data do not include the flowers that were counted during focal observations of strawberry plants.

After 2^nd^ June, we did not conduct any observations of bee behaviour, because of a period of rainy and colder weather. We visited the cages regularly to collect ripe strawberries and noted if there were still any bees alive in the cages (Fig 2).

We harvested strawberries when they were completely red (10^th^ June– 5^th^ July) and we discarded pest-damaged fruits and fruits with broken stems. We weighted the strawberries with a digital balance (Mettler PM200, d = 0.001 grams) immediately after harvest. During strawberry assessments in the lab, we used a protocol that blinded the data collector to cage and treatment of the strawberries. We distinguished between primary, secondary, tertiary, and quaternary strawberries, based on the sequence in which they had flowered on the stalk.

In November, when the bee offspring had built cocoons, we opened all trap nests and assessed bee nest structure and reproduction. We counted the total number of holes used for nesting, as well as the number of holes that had been sealed with a mud plug. We counted the number of cocoons and weighed each of them individually. We determined the survival and sex of the offspring and collected remnants of pollen that the larvae had not consumed, when available (two clothianidin cages and four control cages). In all other cells, the larvae had consumed all the pollen.

### Clothianidin residues

We used the remaining pollen samples to verify that the clothianidin exposure differed between treatments (Tables 1 and S2). In the beginning of June, we collected leaves from both autumn-sown and spring-sown rapeseed to verify that clothianidin was present in the treated plants and absent from the control plants (Tables 1, 2, S2 and S3). We collected leaves instead of flowers because we avoided entering the cages when the bees were most active, which coincided with the blooming of the autumn-sown rapeseed. The leaves were frozen immediately after collection, whereas pollen was stored at ambient temperature until November 2014, when the nests were opened, and we froze it. We analysed pollen samples and leaves from autumn-sown rapeseed for residues of five neonicotinoids: acetamiprid (limit of detection (LOD) = 0.05 ng/ml), imidacloprid (LOD = 0.1 ng/ml), clothianidin (LOD = 0.5 ng/ml), thiacloprid (LOD = 0.05 ng/ml), thiamethoxam (LOD = 1 ng/mg) (Tables 1 and S2). In this analysis, the exact concentration of leaf matter is unknown, but it was similar for all samples. Concentrations can thus only be used to verify differences between the two treatments, or among cages and should not be compared with concentrations in other studies. The leaf samples from two of the cages (one control and one clothianidin) were handled together with clothianidin in the lab and showed extremely high concentrations of clothianidin (S2 Table). We therefore expect them to have been contaminated in the lab. In 2022, we analysed the clothianidin content (LOD = 0.003 ng/g) of leaves from spring-sown and autumn-sown rapeseed (Tables 2 and S3). However, since there were no remaining samples of autumn-sown rapeseed from the two cages where previous samples were expected to have been contaminated, we could not verify the clothianidin treatment in these two cages. We therefore ran all statistical analyses with and without these two cages, which had no qualitative impact on the results (S4 Table).

**Table 1:** Neonicotinoid residues in collected samples.

				Control		Clothianidin	
	LD50 (μg/bee)	LOD	Substrate	Sampled cages (n)	Conc. ng/g (range)	Sampled cages (n)	Conc. ng/g (range)
**Acetamiprid**	7.07	0.03	Pollen	4	< LOD	2	<LOD—0.1
	7.07	0.05	Leaves	5	< LOD	5	< LOD
**Clothianidin**	0.02	0.5	Pollen	4	< LOD	2	1.7–1.8
	0.02	0.5	Leaves	5	< LOD	5	2.4–6.5
**Imidacloprid**	0.02	0.2	Pollen	4	< LOD	2	< LOD
	0.02	0.1	Leaves	5	< LOD	5	< LOD
**Thiacloprid**	14.6	0.02	Pollen	4	<LOD—0.4	2	<LOD—0.7
	14.6	0.05	Leaves	5	< LOD	5	< LOD
**Thiamethoxam**	0.03	0.15	Pollen	4	< LOD	2	< LOD
	0.03	1	Leaves	5	< LOD	5	< LOD

The table shows the concentration (range of detected substances in the samples), the LD_50_ (50% mortality, 24 h after topical application) for honey bees [32], the limit of detection (LOD) and the number of samples analysed. LOD and detected concentrations are given in ng/g for pollen and ng/ml for leaves. Clothianidin was detected in all samples from clothianidin cages, but not in any of the control cages. The exact concentration of leaf matter in the analysed solution is unknown, but similar among samples and can therefore only be used to verify the difference between treatments or among cages, but not with detected concentrations in other studies. Leaves in this analysis were from autumn-sown rapeseed. Pollen was sampled from the nests and may consist of spring-sown rapeseed, autumn-sown rapeseed, strawberry, dandelion, or a mixture of these. In most nests, the larvae had consumed all the pollen, which is why only six cages (four control and two clothianidin) were sampled. In this table, we have removed two samples that we believe were contaminated in the lab. For detailed data, including these two cages, see S2 Table.

**Table 2:** Clothianidin residues in rapeseed leaves of autumn-sown and spring-sown rapeseed.

	Control		Clothianidin	
Variety	Sampled cages (n)	Conc. ng/g (range)	Sampled cages (n)	Conc. ng/g (range)
Visby (autumn-sown)	5	< LOD	5	0.01–0.08
Majong (spring-sown)	6	< LOD	6	< LOQ (>LOD)– 0.01

Clothianidin residues in collected samples from the second analysis, which were stored in a -20 freezer from 2015 to 2022. The limit of detection (LOD) was 0.003 ng/g and the limit of quantification (LOQ) was 0.005 ng/g. Clothianidin was detected in all samples from clothianidin cages (> LOD), but in one sample it was below the limit of quantification (< LOQ). Clothianidin was not detected in any of the samples from control cages (< LOD). For autumn-sown rapeseed, samples were lacking in two cages. In this analysis, the concentrations of the leaf matter was known and concentrations are the true concentrations of the leaves. Detailed data per cage are presented in S3 Table.

### Statistical analyses

We analysed the data using R version 4.1.0 and used Bayesian logistic models (package ‘MCMCglmm’[33]). In all models, we specified clothianidin treatment as a fixed factor. Additional test details are summarized in Table 3. All raw data are available in S1 Data.

**Table 3:** Summary of the statistical analyses.

Dependent variable	Additional explanatory factor	Error distribution	Aggregation level	Random factor
Time per foraging trip (ln-transformed)	-	-	Individual foraging trip	Cage
Flowers per foraging trip (ln-transformed)	-		Individual foraging trip	Cage
Time per 10 visits to rapeseed flowers (ln-transformed)	-	Gaussian	Individual foraging trip	Cage
Visits per strawberry flower and minute (ln-transformed)	-	Gaussian	Day and cage (mean)	Cage
Strawberry weight (ln-transformed and scaled within flower sequence)	Ripening date (scaled)	Gaussian	Individual strawberry	Cage + Pot
Number of cocoons	-	Poisson	Cage (sum)	-
Cocoon weight	Sex	Gaussian	Individual cocoon	Cage
Offspring sex (female/male)	-	Categorical	Individual cocoon	Cage
Presence of outer mud plug (yes/no)	-	Categorical	Individual hole	Cage

As a first option, we analysed the data at the finest resolution, without any transformations, and specified a residual distribution that fitted the distribution and type of data. When data included repeated measurements per cage, we specified cage id as a random factor to account for the non-independence of these measurements. For strawberries, each pot was defined as an additional random factor. When data were zero-inflated, we reduced zero-inflation by aggregating data per day. Because MCMCglmm does not give the option of specifying Gamma or negative binomial distribution, we ln-transformed the data and assumed Gaussian distribution of residuals in cases where any of these distributions would have been appropriate.

We used a few explanatory variables in addition to clothianidin treatment in some models. For cocoon weight, we added sex to account for the fact that females are larger than males. For strawberry weight, we specified an interaction between treatment and normalized (mean = 0, sd = 1) harvest date, to assess if the effect of clothianidin increased over time. Strawberry flowers on the same branch open in a specific order, with the first (primary flower) having the highest number of pistils and usually developing into the largest strawberry. To take this variability into account, while minimizing the complexity of the model and avoiding collinearity with harvest date, we normalized weight (mean = 0, sd = 1) within each flower sequence. Before doing this, we ln transformed weight to allow proportional comparisons of strawberries from clothianidin and control cages.

## Results

### Pollination services

**Fig 3:**
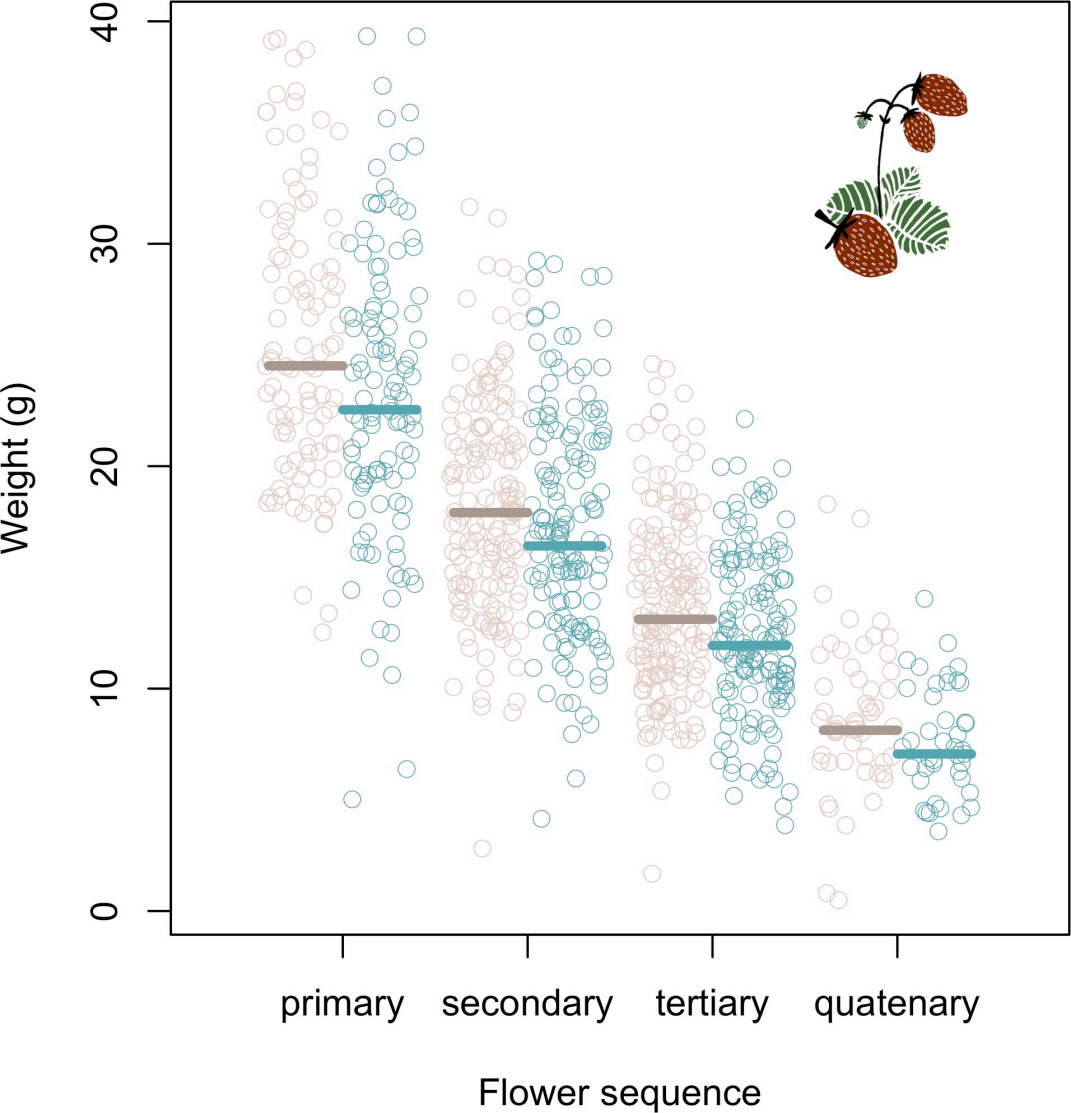
Strawberries weighed less in cages where the bees had been exposed to clothianidin via seed-coated rapeseed. Circles show raw data and lines show back-transformed posterior means from the model for control (beige circles, brown lines) and clothianidin (turquoise circles and lines) cages. Weight is shown separately for strawberries from each of the four flower sequences.

In total, we weighed 925 strawberries. The strawberries weighed between 8% (primary strawberries) and 13% (quaternary strawberries) less in clothianidin cages than in control cages (Table 4, Fig 3). Strawberry weight declined over time (CL_low_ < mean < CL_high_ = -0.25 < -0.20 < -0.14, p < 0.001), but the difference between the two treatments was unaffected by time (treatment × harvest date: CL_low_ < mean < CL_high_ = -0.15 < 0.04 < 0.07, p = 0.42). Females of *O*. *bicornis* were responsible for 90.1% ± 12.9 (raw mean ± sd) of the visits to strawberry flowers. When conducting focal observations of strawberry plants, we found that the flowers received 0.08 ± 0.04 (raw mean ± sd) visits per minute independently of treatment (Table 4).

**Table 4:** Summary of the results from statistical analyses.

Dependent variable	Post. estimate intercept	Post. estimate clothianidin	Lower 95% CI clothianidin	Upper 95% CI clothianidin	*p*
Time per entire foraging trip	5.07	-0.12	-0.46	0.23	0.50
Flowers per entire foraging trip	3.40	-0.25	-0.65	0.18	0.30
Time per 10 visits to rapeseed flowers	3.78	0.09	0.02	0.16	< 0.002
Time per 10 visits to rapeseed flowers (excluding outliers)	3.78	0.06	0.005	0.12	0.02
Visits to strawberry flowers (ln)	-2.78	-0.01	-0.48	0.47	0.95
Strawberry weight (ln-transformed and scaled within flower sequence)	0.14	-0.28	-0.54	-0.03	0.03
Number of cocoons	2.37	-0.21	-0.79	0.32	0.34
Cocoon weight	0.07	0.005	-0.002	0.01	0.17
Sex ratio	105.31	-50.69	-141.30	27.87	0.17
Presence of outer mud plug	-816.19	637.32	53.76	1404.33	0.002

We present untransformed posterior estimates of the intercept and treatment, as well as the 95% credibility intervals (95% CI) and p values for the treatment effect. The results from statistical analyses where we excluded the two cages in which we were unable to verify the clothianidin content are presented in S3 Table. Exclusion of these two cages had no qualitative impact on the results.

### Foraging efficiency

**Fig 4:**
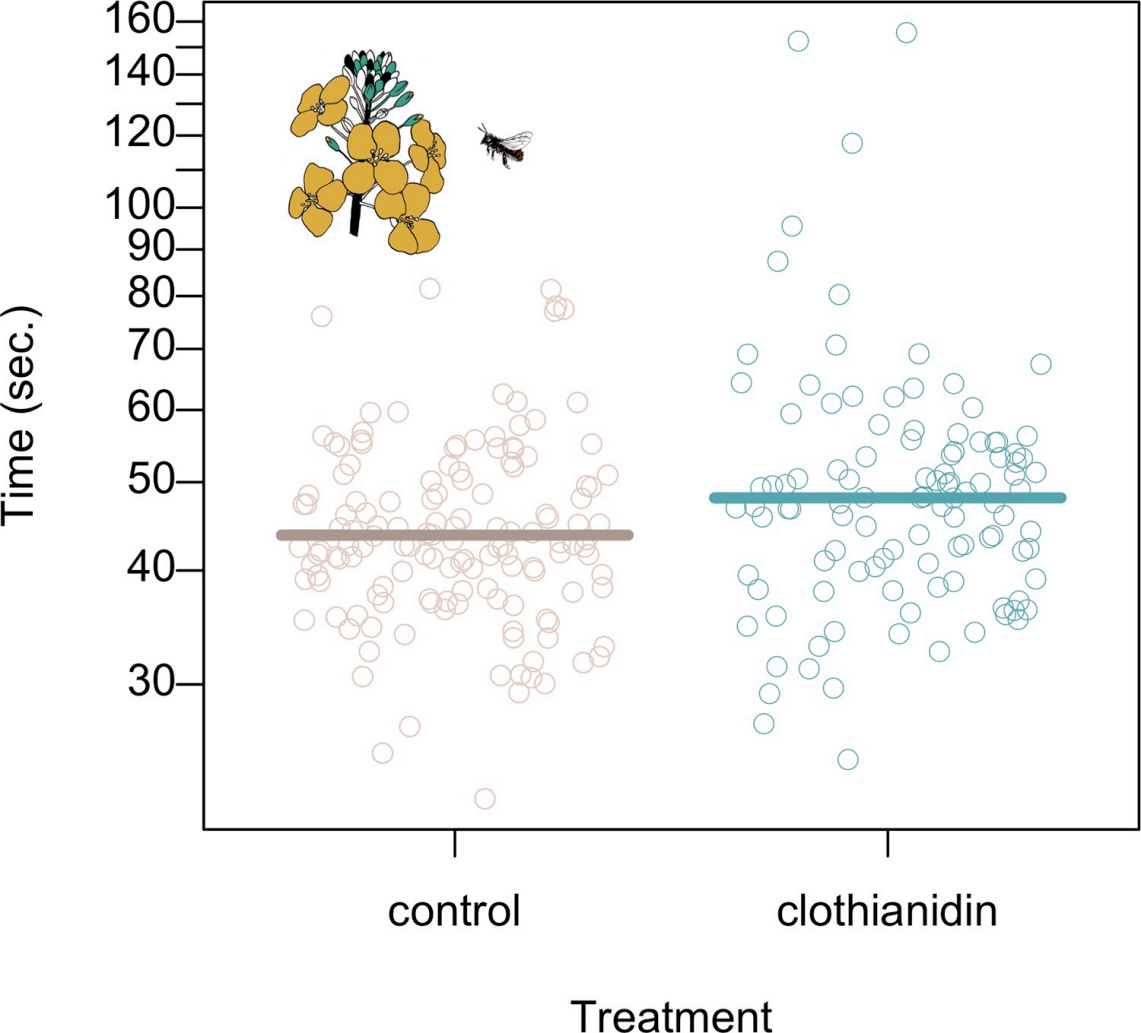
Bees foraging from rapeseed that had been seed-coated with clothianidin used more time to visit ten rapeseed flowers. Dots show raw data and lines show back-transformed posterior means. Exclusion of three outliers had no impact on the result (Table 4).

During the early stage of the experiment, we observed 88 entire foraging trips (between six and 11 trips per cage), where females were observed from when they left the trap nest to when they entered it again. Apart from foraging, these trips also included other activities such as resting and collection of mud for the nests. During six of the trips, the bees visited a few flowering dandelions (mean = 6, median = 2), but most of the visits were to the crop flowers. Trip duration was on average 213.5 ± 46.7 sec and included 45.6 ± 19.7 (raw mean ± sd) flowers. There was no difference between treatments (Table 4). During the late stage of the experiment, we recorded 254 flights where females visited 10 rapeseed flowers in a row. According to the back-transformed model estimates, this took the bees 43.8 sec in control cages and 10.2% longer in clothianidin cages (Table 4, Fig 4). To verify that this result was not driven by three outliers where clothianidin-exposed bees took more than 115 sec to visit 10 flowers, we repeated the analysis without these three data points, which had no qualitative impact on the results (Table 4).

### Nest building and offspring production

Lack of an outer mud plug was more frequent in clothianidin cages than in control cages (Table 4). In five out of six clothianidin cages, at least one hole was unsealed (in total nine out of 25 holes), whereas in control cages only a single hole in one cage was unsealed (in total one out of 32 holes). Females produced on average 10.3 ± 5.0 (raw mean ± sd) cocoons per cage, of which 34.1% ± 19.0 (raw mean ± sd) were females. These numbers were unaffected by the clothianidin treatment (Table 4). The offspring weighed on average 89.7 mg ± 16.7 (raw mean ± sd) and there was no difference between treatments (Table 4). We did not test if the treatment affected offspring survival rate, because only four (one from a clothianidin cage and three from control cages) of the 111 larvae died.

## Discussion

The aim of this study was to assess if and how the exposure to clothianidin via seed-treated rapeseed affected the behaviour, pollination performance (to strawberry) and reproduction of the solitary bee *O*. *bicornis*. We expected clothianidin-exposed bees to be less active, visit fewer flowers and die earlier, resulting in impaired pollination (estimated as lower strawberry weight) and reduced bee reproduction.

Indeed, clothianidin-exposed bees used 10% more time to conduct ten visits to rapeseed flowers. This could either reflect higher availability of floral resources [34], as a potential consequence of reduced pest pressure due to the clothianidin treatment, or distorted foraging behaviour of clothianidin-exposed bees [35]. Since we did not observe any pollen beetles, or other evident pest insects in the cages, we suggest the latter explanation, which is in line with the previous finding that clothianidin prolongs the time that females of *O*. *bicornis* spend on visiting and searching for flowers [35]. We did not observe a similar difference regarding the length of entire foraging trips, or the number of visited flowers during these trips. It is, however, conceivable that this proxy for foraging efficiency is more prone to stochastic variation (these trips also included resting and mud collection), reducing the statistical power to detect any effect, or that effects on bee activity were not yet detectable during the early phase of the experiment when these data were collected. Because it was time consuming to collect data on entire foraging trips, we also collected less of these data (n = 88) than on ten visits to rapeseed flowers (n = 254), resulting in lower statistical power and reduced probability of detecting existing differences. Therefore, we cannot tell if the difference between the early and late measurements is caused by methodological differences between our estimates of forage efficiency, or if the bees were able to compensate for reduced foraging efficiency, by resting less, or collecting less mud.

Strawberries weighed around 10% less in clothianidin cages than in control cages, suggesting that clothianidin-exposed bees performed worse as pollinators than unexposed bees. This is in line with a previous study, where exposure to a neonicotinoid reduced pollination performance in bumblebees [17, but see 18]. Surprisingly, the lower activity that we observed on rapeseed flowers could not be extrapolated to strawberry flowers. Instead, strawberry flowers received similar number of visits in the two treatments, and we were thus unable to relate the lower strawberry weight to reduced foraging activity. Although it is possible that we failed to capture long-term effects on flower-visitation of strawberries (caused by increasing effects on bee inactivity throughout the experiment, or reduced bee longevity), which we recorded before we observed foraging efficiency on rapeseed (Fig 1), the lower weight of strawberries in clothianidin cages was manifest throughout the experiment (i.e., it was unrelated to harvest date, see Results). This indicates that another pollination-related mechanism reduced the pollination success, such as reduced propensity to collect pollen, which has indeed been observed in bumblebees after exposure to the neonicotinoid thiamethoxam [36]. Since we were unable to identify the mechanism by which bees potentially affected pollination by strawberry flowers *per se*, our results need to be treated with care.

By the end of the study, we observed that more than a third of the nest holes in clothianidin cages were unsealed (compared to 1/32 in control cages). Because the outer plug is expected to protect the offspring against parasites and unfavourable climate [37–39], reduced propensity to seal the nests could have serious implications for offspring fitness under field conditions. However, because recent studies have shown that neonicotinoids can delay egg-laying in *Osmia sp*. [40, 41], it is also possible that egg-laying and nest construction had ended in control cages, when the period of rainy weather started, while it occurred later in the clothianidin cages such that the bees were interrupted before sealing the last nests. It is therefore possible that reduced propensity to seal the nests was an outcome of the combined effects of delayed egg-laying (due to clothianidin exposure) and the specific conditions for this experiment rather than a direct effect of clothianidin on bee behaviour.

We found no effect of clothianidin on offspring number, size, or sex, despite that the concentrations we observed in pollen were within the range where clothianidin is expected to have adverse effects on the reproduction of *O*. *bicornis* [5]. Previous studies on how neonicotinoids, including clothianidin, affect the reproduction of *O*. *bicornis* show varying results. While there is solid evidence that neonicotinoids can reduce solitary bee reproduction [5–6, 42], other studies have failed to identify such effects [e.g. 43, 44]. It is possible that this divergence among studies depends on whether the bees additionally suffer from limited access to a diverse diet [42], and whether they need to fly and navigate [8], handle flowers of high complexity [45], and learn new skills [46]. In our study, adverse effects of clothianidin on reproduction may have been mitigated because the bees did not have to navigate through complex landscapes, or learn how to handle flowers of high complexity. Another likely explanation is that the bees suffered from limited access to pollen and nectar, so that reproduction instead was strongly limited by forage availability [47] and any potential effect of clothianidin may have been overshadowed.

Some of the diverging results from previous studies [e.g. 6 and 43] likely reflect a variation in exposure rates among studies, which influences how neonicotinoids affect *O*. *bicornis* under field conditions [5]. This underlines the importance of using field-realistic exposure rates in cage studies, if the aim is to get results that are relevant for field conditions. While we indeed exposed the bees to clothianidin via seed-treated rapeseed, which is a common field scenario, it is still possible that the exposure did not reflect field conditions. First, when other flowers are available, *O*. *bicornis* collect little pollen from rapeseed [6, 47–49]. However, rapeseed is likely an important nectar source for adult bees, even when they mainly collect pollen from other plants [49, 50], and because neonicotinoids are systemic and long-lived, they disperse to the surrounding vegetation [6, 51, 52], where bees could be exposed also when foraging on untreated plants. The provision of both autumn and spring-sown plants, to extend the flowering period, may also not reflect field conditions, where bees would normally forage from either one or the other. On the other hand, the concentrations in the two varieties were of similar magnitude (Table 2) and the extremely high number of plants in a real rapeseed field would likely result in an extended flowering period compared to the short period of the few autumn-sown rapeseed plants used in our study.

Additional stressors, such as potential exposure to pyrethrin during the early phase of the experiment, or competition from a few spontaneously occurring bee males, may have impacted the bees, and interfered with the results. We cannot exclude that these factors increased the unexplained variation among cages (resulting in underestimation of effects) or made the bees more sensitive to the clothianidin treatment (resulting in overestimation of effects), but they were not biased towards one or the other of the treatments. Another, potentially more important, factor in our experiment, is the low number of cages (n = 12), making the results sensitive to random variations unrelated to the clothianidin treatment, but also reduces the statistical power to detect effects.

To summarize, we observed that foraging speed, pollination performance (estimated as strawberry weight), and the propensity to seal the nest holes differed between clothianidin and control cages. The effects that we observed on bee behaviour appeared during the late part of the experiment, suggesting either delayed response, negligible effects of short-term exposure, or higher sensitivity of our behavioural protocol used during the late period. As we failed to establish a mechanistic link in the form of visitation rate, or behaviour on strawberry flowers, we cannot confirm that the reduced strawberry weight was a direct result from modified bee behaviour caused by clothianidin exposure. Yet, the lower strawberry weight in clothianidin cages supports previous findings that neonicotinoid exposure can reduce bees’ pollination performance [17, but see 18] and highlights the need to explore indirect effects of plant protection products, with impacts beyond directly exposed organisms.

## Supporting information

S1 Table Number of females per cage that were manually released from the cocoons. (DOC)

S2 Table Detailed information about neonicotinoid residues in pollen collected by the bees, and leaves from the autumn sown rapeseed (variety Visby). (DOC)

S3 Table Detailed information about clothianidin residues in leaves from autumn sown (variety Visby) and spring sown (variety Majong) rapeseed. (DOC)

S4 Table Summary of the results, excluding the two cages for which we were unable to verify the clothianidin content of the leaves. (DOC)

S1 Data All raw data supporting the statistical analyses. (XLSX)
