# Supplementary material for: Seed-coating of rapeseed (Brassica napus) with the neonicotinoid clothianidin affects behaviour of red mason bees (Osmia bicornis) and pollination of strawberry flowers (Fragaria × ananassa)
Source: PLoS One. 2022 Sep 8;17(9):e0273851. doi: 10.1371/journal.pone.0273851 (PMC9455870; doi:10.1371/journal.pone.0273851)
Supplement: S1 Table — (DOC) [file pone.0273851.s001.doc]

**S1 Table**

**Seed-coating of rapeseed (*Brassica napus*) with the neonicotinoid clothianidin affects behaviour of red mason bees (*Osmia bicornis*) and pollination of strawberry flowers (*Fragaria × ananassa*)**

Lina Herbertsson^1,2^*, Björn K. Klatt^1,2^,*, Maria Blasi^1^*, Maj Rundlöf^2^ & Henrik G. Smith^1,2^

**Affiliations**

^1^ Lund University, Centre for Environmental and Climate Research, 22362 Lund, Sweden

^2^ Lund University, Department of Biology, 22362 Lund, Sweden

*Corresponding authors, who contributed equally to this work.

**Contact information of corresponding authors:**

Lina Herbertsson, Department of Biology, Lund University, SE-223 62 Lund, Sweden, e-mail: lina.herbertsson@biol.lu.se, phone: +46 70 296 42 55

Björn K. Klatt, Centre for Environmental and Climate Research & Department of Biology, Lund University, SE-223 62 Lund, Sweden, e-mail: bjorn.klatt@biol.lu.se

Maria Blasi, Centre for Environmental and Climate Research, Lund University, SE-223 62 Lund, Sweden, e-mail: maria.blasi_romero@cec.lu.se

| **S1 Table. Number of females per cage that were manually released from the cocoons.** Each cage was provided with four female cocoons and five male cocoons the 25^th^ of April. Thirteen of the females (seven from clothianidin cages and six from control cages) had not emerged the 5^th^ of May and were then manually released. The table shows the number of released females per cage. | | |
| --- | --- | --- |
| **Cage** | **Treatment** | **Number of manually released females** |
| 1 | Clothianidin | 0 |
| 2 | Control | 0 |
| 3 | Clothianidin | 2 |
| 4 | Control | 0 |
| 5 | Clothianidin | 1 |
| 6 | Control | 2 |
| 7 | Control | 1 |
| 8 | Clothianidin | 0 |
| 9 | Control | 2 |
| 10 | Clothianidin | 1 |
| 11 | Control | 1 |
| 12 | Clothianidin | 3 |
