# Supplementary material for: Seed-coating of rapeseed (Brassica napus) with the neonicotinoid clothianidin affects behaviour of red mason bees (Osmia bicornis) and pollination of strawberry flowers (Fragaria × ananassa)
Source: PLoS One. 2022 Sep 8;17(9):e0273851. doi: 10.1371/journal.pone.0273851 (PMC9455870; doi:10.1371/journal.pone.0273851)
Supplement: S2 Table — (DOC) [file pone.0273851.s002.doc]

**S2 Table**

**Seed-coating of rapeseed (*Brassica napus*) with the neonicotinoid clothianidin affects behaviour of red mason bees (*Osmia bicornis*) and pollination of strawberry flowers (*Fragaria × ananassa*)**

Lina Herbertsson^1,2^*, Björn K. Klatt^1,2^,*, Maria Blasi^1^*, Maj Rundlöf^2^ & Henrik G. Smith^1,2^

**Affiliations**

^1^ Lund University, Centre for Environmental and Climate Research, 22362 Lund, Sweden

^2^ Lund University, Department of Biology, 22362 Lund, Sweden

*Corresponding authors, who contributed equally to this work.

**Contact information of corresponding authors:**

Lina Herbertsson, Department of Biology, Lund University, SE-223 62 Lund, Sweden, e-mail: lina.herbertsson@biol.lu.se, phone: +46 70 296 42 55

Björn K. Klatt, Centre for Environmental and Climate Research & Department of Biology, Lund University, SE-223 62 Lund, Sweden, e-mail: bjorn.klatt@biol.lu.se

Maria Blasi, Centre for Environmental and Climate Research, Lund University, SE-223 62 Lund, Sweden, e-mail: maria.blasi_romero@cec.lu.se

| **S2 Table. Detailed information about neonicotinoid residues in pollen collected by the bees, and leaves from the autumn sown rapeseed (variety Visby).** For leaves, the data presented are concentrations in extractions, where the exact concentration of leaf matter is unknown, but similar among samples. Pollen was only available for two clothianidin cages (3 and 5) and four control cages (6, 7, 9 and 11), whereas leaves were available for all but one cage (11). The clothianidin concentration was always below the limit of detection (LOD) in control cages, except for cage 2. In contrast, we detected clothianidin in all samples taken from clothianidin cages. Because samples from cage 1 and 2 (*) with extremely high concentrations of clothianidin were handled in the lab together with clothianidin (the only samples handled that day), we suspect them to have been contaminated. As we could not verify the true concentrations of clothianidin for these two cages, we have run all the analyses with and without them. NA means that samples are lacking. | | | | | | |  |
| --- | --- | --- | --- | --- | --- | --- | --- |
| **Cage** | **Treatment** | **Substrate** | **Clothianidin (ng/g)** | **Acetamiprid (ng/g)** | **Imidacloprid (ng/g)** | **Thiacloprid (ng/g)** | |
| 1 | Clothianidin | Pollen | NA | NA | NA | NA | |
|  |  | Leaves | *317** | <LOD | <LOD | <LOD | |
| 2 | Control | Pollen | NA | NA | NA | NA | |
|  |  | Leaves | *47** | <LOD | <LOD | <LOD | |
| 3 | Clothianidin | Pollen | 1.8 | <LOD | <LOD | 0.043 | |
|  |  | Leaves | 2.8 | <LOD | <LOD | <LOD | |
| 4 | Control | Pollen | NA | NA | NA | NA | |
|  |  | Leaves | <LOD | <LOD | <LOD | <LOD | |
| 5 | Clothianidin | Pollen | 1.7 | 0.09 | <LOD | 0.65 | |
|  |  | Leaves | 6.5 | <LOD | <LOD | <LOD | |
| 6 | Control | Pollen | <LOD | <LOD | <LOD | <LOD | |
|  |  | Leaves | <LOD | <LOD | <LOD | <LOD | |
| 7 | Control | Pollen | <LOD | <LOD | <LOD | <LOD | |
|  |  | Leaves | <LOD | <LOD | <LOD | <LOD | |
| 8 | Clothianidin | Pollen | NA | NA | NA | NA | |
|  |  | Leaves | 2.4 | <LOD | <LOD | <LOD | |
| 9 | Control | Pollen | <LOD | <LOD | <LOD | 0.12 | |
|  |  | Leaves | <LOD | <LOD | <LOD | <LOD | |
| 10 | Clothianidin | Pollen | NA | NA | NA | NA | |
|  |  | Leaves | 2.7 | <LOD | <LOD | <LOD | |
| 11 | Control | Pollen | <LOD | <LOD | <LOD | 0.37 | |
|  |  | Leaves | NA | NA | NA | NA | |
| 12 | Clothianidin | Pollen | NA | NA | NA | NA | |
|  |  | Leaves | 6.3 | <LOD | <LOD | <LOD | |
