# Supplementary material for: Seed-coating of rapeseed (Brassica napus) with the neonicotinoid clothianidin affects behaviour of red mason bees (Osmia bicornis) and pollination of strawberry flowers (Fragaria × ananassa)
Source: PLoS One. 2022 Sep 8;17(9):e0273851. doi: 10.1371/journal.pone.0273851 (PMC9455870; doi:10.1371/journal.pone.0273851)
Supplement: S3 Table — (DOC) [file pone.0273851.s003.doc]

**S3 Table**

**Seed-coating of rapeseed (*Brassica napus*) with the neonicotinoid clothianidin affects behaviour of red mason bees (*Osmia bicornis*) and pollination of strawberry flowers (*Fragaria × ananassa*)**

Lina Herbertsson^1,2^*, Björn K. Klatt^1,2^,*, Maria Blasi^1^*, Maj Rundlöf^2^ & Henrik G. Smith^1,2^

**Affiliations**

^1^ Lund University, Centre for Environmental and Climate Research, 22362 Lund, Sweden

^2^ Lund University, Department of Biology, 22362 Lund, Sweden

*Corresponding authors, who contributed equally to this work.

**Contact information of corresponding authors:**

Lina Herbertsson, Department of Biology, Lund University, SE-223 62 Lund, Sweden, e-mail: lina.herbertsson@biol.lu.se, phone: +46 70 296 42 55

Björn K. Klatt, Centre for Environmental and Climate Research & Department of Biology, Lund University, SE-223 62 Lund, Sweden, e-mail: bjorn.klatt@biol.lu.se

Maria Blasi, Centre for Environmental and Climate Research, Lund University, SE-223 62 Lund, Sweden, e-mail: maria.blasi_romero@cec.lu.se

**S3 Table. Detailed information about clothianidin residues in leaves from autumn-sown (variety Visby) and spring-sown (variety Majong) rapeseed.** The limit of detection (LOD) was 0.003 ng/g and the limit of quantification (LOQ) was 0.005 ng/g. The content in these samples was analysed in 2022, after seven years in a -20 freezer. No leaf matter was available from autumn sown rapeseed in cages 1 and 2. As we could not verify the concentrations of clothianidin in these two cages, we have run all the analyses with and without them. NA means that samples are lacking.

| **Cage** | **Treatment** | **Variety** | **Clothianidin (ng/g)** |
| --- | --- | --- | --- |
| 1 | Clothianidin | Visby (autumn-sown) | NA |
|  |  | Majong (spring-sown) | *> LOD; <LOQ* |
| 2 | Control | Visby (autumn-sown) | NA |
|  |  | Majong (spring-sown) | *<LOD* |
| 3 | Clothianidin | Visby (autumn-sown) | 0.01 |
|  |  | Majong (spring-sown) | *> LOD; <LOQ* |
| 4 | Control | Visby (autumn-sown) | *<LOD* |
|  |  | Majong (spring-sown) | <LOD |
| 5 | Clothianidin | Visby (autumn-sown) | 0.03 |
|  |  | Majong (spring-sown) | 0.01 |
| 6 | Control | Visby (autumn-sown) | <LOD |
|  |  | Majong (spring-sown) | <LOD |
| 7 | Control | Visby (autumn-sown) | <LOD |
|  |  | Majong (spring-sown) | <LOD |
| 8 | Clothianidin | Visby (autumn-sown) | 0.02 |
|  |  | Majong (spring-sown) | 0.01 |
| 9 | Control | Visby (autumn-sown) | <LOD |
|  |  | Majong (spring-sown) | <LOD |
| 10 | Clothianidin | Visby (autumn-sown) | 0.09 |
|  |  | Majong (spring-sown) | 0.01 |
| 11 | Control | Visby (autumn-sown) | <LOD |
|  |  | Majong (spring-sown) | *<LOD* |
| 12 | Clothianidin | Visby (autumn-sown) | 0.01 |
|  |  | Majong (spring-sown) | 0.01 |
