# Supplementary material for: Seed-coating of rapeseed (Brassica napus) with the neonicotinoid clothianidin affects behaviour of red mason bees (Osmia bicornis) and pollination of strawberry flowers (Fragaria × ananassa)
Source: PLoS One. 2022 Sep 8;17(9):e0273851. doi: 10.1371/journal.pone.0273851 (PMC9455870; doi:10.1371/journal.pone.0273851)
Supplement: S4 Table — (DOC) [file pone.0273851.s004.doc]

**S4 Table**

**Seed-coating of rapeseed (*Brassica napus*) with the neonicotinoid clothianidin affects behaviour of red mason bees (*Osmia bicornis*) and pollination of strawberry flowers (*Fragaria × ananassa*)**

Lina Herbertsson^1,2^*, Björn K. Klatt^1,2^,*, Maria Blasi^1^*, Maj Rundlöf^2^ & Henrik G. Smith^1,2^

**Affiliations**

^1^ Lund University, Centre for Environmental and Climate Research, 22362 Lund, Sweden

^2^ Lund University, Department of Biology, 22362 Lund, Sweden

*Corresponding authors, who contributed equally to this work.

**Contact information of corresponding authors:**

Lina Herbertsson, Department of Biology, Lund University, SE-223 62 Lund, Sweden, e-mail: lina.herbertsson@biol.lu.se, phone: +46 70 296 42 55

Björn K. Klatt, Centre for Environmental and Climate Research & Department of Biology, Lund University, SE-223 62 Lund, Sweden, e-mail: bjorn.klatt@biol.lu.se

Maria Blasi, Centre for Environmental and Climate Research, Lund University, SE-223 62 Lund, Sweden, e-mail: maria.blasi_romero@cec.lu.se

**S4 Table. Summary of the results, excluding the two cages for which we were unable to verify the clothianidin content of the leaves. We present untransformed posterior estimate of intercept and treatment, as well as the 95% credibility interval (95% CI) and p-value for the treatment effect.**

| **Dependent variable** | **Post. mean**  **intercept** | **Post. mean treatment** | **Lower  95% CI treatment** | **Upper 95% CI treatment** | ***p*** |
| --- | --- | --- | --- | --- | --- |
| Time per entire foraging bout (ln) | 5.06 | -0.17 | -0.54 | 0.26 | 0.40 |
| Flowers per entire foraging bout (ln) | 3.40 | -0.33 | -0.92 | 0.09 | 0.17 |
| Time per 10 rapeseed flower visits (ln) | 3.78 | 0.11 | 0.04 | 0.18 | 0.002 |
| Visits to strawberry flowers (ln) | -2.79 | -0.10 | -0.65 | 0.46 | 0.71 |
| Strawberry weight (ln-transformed and scaled within flower sequence) | 0.87 | -0.29 | -0.58 | -0.002 | 0.05 |
| Number of cocoons (poisson) | 2.35 | -0.19 | -1.03 | 0.47 | 0.58 |
| Cocoon weight | 0.07 | 0.004 | -0.004 | 0.01 | 0.33 |
| Proportion females | 122.54 | -56.80 | -176.34 | 26.41 | 0.17 |
| Proportion closed nests | -724.55 | 639.04 | 71.37 | 1431.34 | 0.0009 |
